# Acute bacterial meningitis in children presenting to The Children’s Hospital Lahore before and after pneumococcal vaccine in Pakistan National Immunization Program; A comparison

**DOI:** 10.12669/pjms.332.11891

**Published:** 2017

**Authors:** Attia Bari, Fatima Zeeshan, Aizza Zafar, Hasan Ejaz, Uzma Jabeen, Ahsan Waheed Rathore

**Affiliations:** 1 Attia Bari, DCH, MCPS, FCPS. (Paediatric Medicine). Department of Paediatric Medicine, The Children’s Hospital & The Institute of Child Health (CHICH), Lahore, Pakistan; 2 Fatima Zeeshan, MRCPCH, FCPS. (Paediatric Medicine). Department of Paediatric Medicine, The Children’s Hospital & The Institute of Child Health (CHICH), Lahore, Pakistan; 3 Aizza Zafar, M. Phil Microbiology. Department of Microbiology, The Children’s Hospital & The Institute of Child Health (CHICH), Lahore, Pakistan; 4 Hasan Ejaz, M. Phil, PhD Biotechnology. Department of Microbiology, The Children’s Hospital & The Institute of Child Health (CHICH), Lahore, Pakistan; 5 Uzma Jabeen, FCPS. (Paediatric Medicine). Department of Paediatric Medicine, The Children’s Hospital & The Institute of Child Health (CHICH), Lahore, Pakistan; 6 Ahsan Waheed Rathore, MRCPCH, FRCP. Department of Paediatric Medicine, The Children’s Hospital & The Institute of Child Health (CHICH), Lahore, Pakistan

**Keywords:** Acute bacterial meningitis, *Streptococcus pneumoniae*, Vaccination, PCV

## Abstract

**Objective:**

To describe bacteriological profile, morbidity and mortality of acute bacterial meningitis (ABM) in children and to compare these parameters before and after the introduction of Pneumococcal vaccine in Pakistan National Immunization Program.

**Methods:**

The present descriptive study was conducted at the Department of Paediatric Medicine of The Children’s Hospital Lahore from January 2012 to December 2015. A total of 503 children one month to five years of age admitted with diagnosis of meningitis were included. Complete blood count, CSF cytology, biochemistry, culture sensitivity and blood culture sensitivity were performed.

**Results:**

Frequency of meningitis decreased by 50% in 2013-2015 (199 [2012] vs 304 [2013-2015). Most children in both groups were under one year of age. More neurological complications were seen in the group 2, 20% vs 17%. CSF culture positivity decreased from 12% to 6.6%. *Streptococcus pneumoniae* isolation decreased from 5 (2.5%) in 2012 to 4 (1.3%) in 2013-2015. Refusal to take feed (p=0.002), impaired sensorium (p=<0.001), severe malnutrition (p=0.001), prolonged duration of symptoms (p=<0.001) and incomplete vaccination status (0.005) were associated with mortality. Mortality rate decreased from 20 (10%) in 2012 to 17 (5.6%) in 2013-2015 but more children developed neurological sequelae 2.7% versus 1%.

**Conclusion:**

Acute bacterial meningitis mostly affected children <1 year. Frequency of *Streptococcus pneumoniae* and mortality of meningitis decreased significantly after PCV but more neurological complications developed in those children who were unvaccinated in 2013-2015 compared to 2012.

## INTRODUCTION

Acute bacterial meningitis (ABM) is a severe illness mostly affecting children under the age of five years but people of any age can develop ABM. Despite advances in medical treatment ABM remains an important cause of childhood morbidity and mortality throughout the world.^1^ Neurological sequelae are common in children who suffered from ABM.^2^ Fever, vomiting, poor feeding, convulsions, headache, neck stiffness and altered consciousness are common presentations of meningitis in children.^3^ The diagnosis of central nervous system (CNS) infection is made on examination of cerebrospinal fluid (CSF) and CNS infections can be categorized according to pathogen involved into bacterial, viral, fungal or protozoal.^4^ The exact etiological diagnosis is often not possible, because prior antibiotic therapy, low bacterial load and delay in plating for culture.

In children the pathogens responsible for the most cases of meningitis in developing countries are *Streptococcus pneumoniae* and *Haemophilus influenzae* type b (Hib).^5^ There is almost disappearance of *H. influenza* as a cause of meningitis after the introduction of vaccine. A step forward is the development of Pneumococcal Conjugate Vaccine (PCV), after which ABM has become an uncommon disease in developed countries.^6^ Hospital based studies from South Asia showed that 3.57%-10.58% of all hospitalized children had invasive pneumococcal disease.^7^ Recently in 2012, 10-valent Pneumococcal conjugate vaccine (PCV10) is introduced in Expanded Program of Immunization (EPI) in Pakistan.^8^

We therefore conducted the study with the aim to describe bacteriological profile, complications and mortality of acute bacterial meningitis in children and to compare these characteristics of the disease before and after the introduction of PCV in our national immunization program.

## METHODS

This was a prospective longitudinal hospital based descriptive study, conducted at The Children’s Hospital Lahore over a period of four years from January 2012 to December 2015. All those children (1 month-5 years) who were admitted to General Medical Ward with diagnosis of meningitis on the basis of clinical suspicion and underwent lumbar puncture were included.

Inclusion criteria for suspected meningitis as per WHO, was defined as a child aged >1 month to < 5 years with sudden onset of fever (>38.5°C rectal or 38.0°C axillary) and one of the following signs: neck stiffness or flaccid neck, bulging fontanelle (in children aged <12 months), irritability, drowsiness or convulsion along with CSF showing > 10 WBC. Children with co-morbidities like meningomyelocele, hydrocephalus, acute head trauma and prior central nervous system diseases were excluded. Each patient underwent a detailed history and thorough clinical examination. Relevant laboratory data in all patients including complete blood count, CSF cytology, biochemistry, culture sensitivity (C/S) and blood culture sensitivity (C/S) were performed. Seizures, motor or sensory loss, altered sensorium or any neurological deficit was checked during daily examination. CT scan brain was done in patients having neurological complication. Outcome of patients was noted in form of discharged, died, left against medical advice (LAMA) or shifted to other ward like neurosurgery ward for management of any complication.

The data was divided into two groups; Group 1: Patients enrolled from January 2012- December 2012, Group 2: Patients enrolled from January 2013- December 2015. Both groups were compared. For statistical analysis Statistical Package for Social Sciences (SPSS) version 20 software was used.

## RESULTS

During this four-year study period total 503 patients were hospitalized with ABM. Out of 503 children, there were 199 cases in 2012 and 304 in 2013-2015. Frequency of meningitis decreased to half from 200 cases/ year to 100 cases/year. Maximum numbers of children were in one month to one year of age group 136 (68.3%) and 189 (62%) in both groups. Compared to 2012, relatively more (9.5% vs 5%) children in 2013-2015 were between 3-5 years. (Table-I).

**Table-I:** Demographics of children with acute bacterial meningitis.

*Category*	*Group 1 Jan 2012-Dec 2012 Total n = 199 (100%)*	*Group 2 Jan 2013-Dec 2015 Total n=304 (100%)*
***Age***		
Mean age in months	11.33±12	13.66±19
< 1 year	136 (68.3%)	189 (62.2%)
1 year-3 years	53 (26.6%)	86 (28.3%)
3.1 years-5 years	10 (5.0%)	29 (9.5%)
Sex (M:F)	1.7:1	1.6:1
Male	127 (64%)	189 (62%)
Female	72 (36%)	115 (38%)
***Vaccination Status***		
Vaccinated according to EPI	90 (45.2%)	92 (30.3%)
Un-vaccinated	96 (48.2%)	178 (58.5%)
Partially vaccinated	13 (6.6%)	34 (11.2%)
***Severe malnutrition***		
Present	46 (23.1%)	35 (11.5%)
Absent	153 (76.9%)	269 (88.5%)
***Complications***		
No complication	165 (82.9%)	245 (80.6%)
Seizures after 4 days of hospital stay	22 (11%)	38 (12.5%)
Subdural effusion	1 (0.5%)	4 (1.3%)
Hydrocephalus	2 (1%)	11 (3.6%)
Motor loss	9 (4.5%)	4 (1.3%)
Ventriculitis	0	2 (0.7%)
***Out come***		
Discharged	165 (82.9%)	267 (87.8%)
Died	20 (10.1%)	17 (5.6%)
Left against medical advice (LAMA)	12 (6.0%)	12 (3.9%)
Shifted to neurosurgery ward	02 (1%)	8 (2.7%)

The most common presenting symptom was fever (n=190; 95% vs n=291; 95.7%), (n=174; 87% vs n=260; 85.5%) had seizures, (n=50; 25% vs n=91; 30%) presented with poor feeding and (n=46; 23% vs n=107; 35%) had impaired consciousness in 2012 vs 2013-2015 respectively. In 2012 presentation with refusal to take feed (p=0.008) and with impaired conscious state were independent predictors of death (p=0.002). CSF culture positivity, severe malnutrition and development of complications also had statistically significant p values of 0.039, 0.037 and <0.001 respectively. In 2013-2015 there were almost similar statistically significant results (Table-II).

**Table-II:** Factors associated with outcome of children with acute bacterial meningitis in 2013-2015.

*Characteristics*	*Outcome*				*p-value*

	*Discharged n= 267*	*Died n= 17*	*LAMA n=12*	*Shifted n=8*
***Altered Mental Status at presentation***				
Yes	81 (75.8%)	13 (12%)	6 (5.6%)	7 (6.5%)	<0.001
No	186 (94.5%)	4 (2%)	6 (3%)	1 (0.5%)
***Poor Feeding***				
Yes	72 (79%)	11 (12%)	3 (3.4%)	5 (5.5%)	0.002
No	195 (91.5%)	6 (2.8%)	9 (4.2%)	3 (1.4%)
***Severe malnutrition***				
Yes	24 (68.5%)	6 (17%)	2 (5.7%)	3 (8.5%)	0.001
No	243 (90.3%)	11 (4%)	10 (3.7%)	5 (1.8%)
***Complications***				
Yes	34 (61.8%)	11 (20%)	2 (3.6%)	8 (14.5%)	<0.001
No	233 (93.5%)	6 (2.4%)	10 (4%)	
***Duration of symptoms***				
1-2 days	157 (90.7%)	7 (4%)	8 (4.6%)	1 (0.6%)	<0.001
3-7 days	96 (89.7%)	7 (6.5%)	2 (1.9%)	2 (1.9%)
> 7 days	14 (58.3%)	3 (12.5%)	2 (8.3%)	5 (20.8%)
***Vaccination***				
Complete	88 (96%)	1 (1%)	1 (1%)	2 (2%)	0.005
Incomplete	24 (70.5%)	5 (14.7%)	2 (6%)	3 (8.8%)
Not done	155 (87%)	11 (6.3%)	9 (5%)	3 (1.6%)

Uneventful course was present in (n=165; 83% vs n=245; 80.6%). There were significantly more complications in 2013-2015 group (n=59; 20%) vs (n=34; 17%). Type of complications are described in (Table-I).

The blood culture (12.6%vs 6%) and CSF culture positivity was (12% vs 7%) in 2012 vs 2013-2015 group respectively. The common etiology based on CSF culture is shown in (Table-III). *Coagulase negative Staphylococci* (C*oNS*) was the predominating organism 16 (8%) isolated on blood culture followed by *Pseudomomas* 3 (1.5%), *Streptococcus pneumoniae* 2 (1%), *Acinetobacter* 2 (1%), *Klebsiella* and *Staphylococcus aureus* 1 (0.5%) in 2012. In 2013-2015 organism isolated were (C*oNS*) 12 (4%), *Staphylococcus aureus* 1 (0.3%), *Klebsiella* 2 (0.7%)*, Pseudomomas* 2 (0.7%) *and Citrobacter* 1 (0.3%).

**Table-III:** CSF culture isolates in children with acute bacterial meningitis (2012-2015).

*CSF Culture*	*Duration*		

*Isolates*	*Group 1 Jan 2012- Dec 2012 n=199*	*Group 2 Jan 2013- Dec 2015 n= 304*	*Total in 4 Years Jan 2012- Dec 2015 n= 503*
CoNS	11 (5.5%)	3 (1%)	14 (2.7%)
S.pneumoniae	5 (2.5%)	4 (1.3%)	9 (1.7%)
H.influenzae	2 (1%)	0 (0%)	2 (0.4%)
S.pyogenes	2 (1%)	0 (0%)	2 (0.4%)
E. coli	2 (1%)	1 (0.3%)	3 (0.6%)
S. aureus	1 (0.5%)	2 (0.7%)	3 (0.6%)
Klebsiella	1 (0.5%)	7 (2.3%)	8 (1.5%)
Pseudomonas	0	2 (0.7%)	2 (0.4%)
Citrobacter	0	1 (0.3%)	1 (0.2%)
Total Positivity	24 (12%)	20 (6.6%)	44 (8.7%)

Outcome of children is shown in Table-I. Mortality rate decreased from 20 (10%) in 2012 to 17 (5.6%) in 2013-2015 but more children with neurological complications were shifted to neuro-surgery for management in 2013-2015 as compared to 2012 (n=8; 2.6% vs n=2; 1%).

## DISCUSSION

Worldwide ABM remains a disease with devastating consequences particularly in unvaccinated children and it can cause fatal outcome or severe neurological sequelae especially when there is delay in diagnosis and antibiotic administration. World Health Organization (WHO) estimates that each year, half a million children under 5 years are killed by *Streptococcus pneumoniae* and most of these deaths occurring in developing countries.^9^ The introduction of conjugate vaccines have had a major impact on characteristics and epidemiology of ABM. PCV was introduced in Pakistan by the end of 2012 to reduce this disease burden.

The disease pattern, bacteriology and mortality associated with ABM were investigated in this study and comparison was done for the children presented before and after introduction of PCV in Pakistan EPI program. The demographic analysis showed a high proportion of patients having male gender (63%). This may signify the male dominance and sex discrimination in South East Asia. A preponderance of males 1.5:1 was noted in an Indian study and in various other studies showing male preponderance.^1^,^10^

In our study, 136 (68.3%) and 189 (62%) of our patients were below one year of age confirming that meningitis mostly affect young children as compared to older children. Similar results of meningitis incidence with young age were found in a study published in Niger Med J.^1^ Our 23% and 11% children were severely malnourished consistent with the results of a study done by Khan et al which showed grade III malnutrition in 27% of children signifying their immune deficient state.^11^

Microbial diagnosis of meningitis based on CSF culture was confirmed in only 24 (12%) and 20 (6.6%) of patients in both groups. Our results were comparable with, studies done by Nhantumbo and Rajani in which CSF culture showed positivity in 7.3% and 7.2% respectively.^12^,^13^ A much higher percentage of 62.7% CSF culture positivity was reported in another study published in J Neurosci Rural Practice.^3^ A much high proportion (88% and 93.4%) of all meningitis cases in our study grew no bacterial pathogen. A number of previous studies are consistent with this finding suggesting that the negative cultures are not due to limitations in routine microbiology laboratory procedures.^1^,^14^ The high incidence of sterile CSF may be due to widespread use of antibiotics.

Our study identified *CoNS as* the most common organism isolated 11/199 (5.5%) followed by *Streptococcus pneumoniae* 5/199 (2.5%), *H. influenzae* and *Streptococcus pyogenes in 2012*. In 2013-2015 the commonest organisms were *Klebsiella* 7(2.3%). *Streptococcus pneumoniae 4/304 (1.3%)* and *CoNS* 3/304 (1%) Considering maximum number of CSF cultures positive for *CoNS* in 2012, it may be due to skin contamination which was reduced in later years with appropriate sterilization techniques. Second commonest pathogen isolated in our study was *Streptococcus pneumoniae* and this was consistent with a Korean study in which *Streptococcus pneumoniae* was the commonest pathogen isolated.^15^ Our 2013-2015 isolates results were consistent with a study done by Yadhav in which *Klebsiella* was the commonest organism in CSF isolates.^16^ Literature reviews describing bacteria isolated from children with ABM suggest that both Gram-positive as well as Gram-negative organisms may cause CNS infection.^16^,^17^

In our study acute neurological complications were noted in 34(17%) and 59(19.4%) of our patients. Similar results were seen with 24% complications rate in a study published in Arch Dis Child.^18^ A few studies reported a much higher percentage of complications as compared to our study (39%, 42%).^3^,^10^

Our case-fatality rate was 10.1% and 5.6% close to that (9.4%) reported in a Korean study and 13.9% in an Indian study.^11^,^15^ Higher case fatality rates (19%. 27.2%) were described in various studies.^3^,^18^

## CONCLUSION

The frequency and mortality due to ABM was observed mostly under one year of age and the commonest bacterial pathogen isolated in these cases was *Streptococcus pneumoniae*. Frequency of *Streptococcus pneumoniae* and mortality of meningitis decreased significantly after introduction of PCV in EPI of Pakistan but more neurological complications developed in those children who were unvaccinated in 2013-2015 compared to 2012. Up-to-date vaccination, early referral and timely appropriate treatment can reduce the morbidity and mortality associated with bacterial meningitis.

### Authors’ Contribution

***AB:*** Main author.

***FZ &UJ:*** Data organization, SPSS data compilation, proof reading.

***AZ:*** Facilities and technical support for microbiology.

***HE:*** Performed microbiological experimental work and manuscript editing.

***AWR:*** Final approval of the version to be published.
